# Principal component analysis: development and initial validation of the mirror effects inventory

**DOI:** 10.1186/s40359-023-01397-8

**Published:** 2023-10-31

**Authors:** Wing W. Y. Ho

**Affiliations:** School of Education and Languages, Hong Kong Metropolitan University, Good Shepherd Street, Ho Man Tin, Kowloon, Hong Kong SAR, China

**Keywords:** Mirror effects inventory, Principal component analysis, Counseling education, Qualitative analysis, Quantitative analysis, Thematic analysis

## Abstract

**Background:**

Mirroring is a specific phenomenon that occurs in groups. People observe others enacting different roles in given situations, whereby concretizing the role of the observing aspect of their self as if looking in a mirror. Individuals could acquire emotional, cognitive, imaginative, behavioral, and interpersonal experiences through this process of mirroring; that is, “mirror effects.” Given that there are very few comprehensive scales capable of evaluating mirror effects, the Mirror Effects Inventory (MEI) was developed to measure experiences after participating in counseling education. This study describes the development of the MEI based on an interpersonal perspective and aimed to validate this scale.

**Methods:**

The development and validation of the MEI involved a seven-stage process that utilized both qualitative and quantitative methods. Former students who studied counseling (*N* = 24) were surveyed via semi-structured telephone interviews. A subjective perceived scale (the MEI) was then constructed. The MEI was administered to part-time university students (*N* = 221) studying counseling courses. Psychometric tests were performed to examine its validity and reliability. Validation of the MEI included examining correlations between three types of mirror effects and age differences and educational attainment.

**Results:**

The PCA revealed three types of mirror effects: general, positive, and negative. These components accounted for 53.82% of the total variance. Reliability estimates measured using Cronbach’s alpha of .88 indicated that the items were internally consistent. The findings showed that as the general mirror effect increased, there was a corresponding increase in the positive mirror effect and a decrease in the negative mirror effect. However, an increase in positive mirror effects was associated with an increase in negative mirror effects.

**Conclusions:**

The development of a reliable and valid measure of mirror effects has benefits and implications for counseling education and clinical practice. Further empirical research could contribute to resolving the controversies concerning the basic conceptual understanding of MEI.

**Supplementary Information:**

The online version contains supplementary material available at 10.1186/s40359-023-01397-8.

## Background

Numerous self-report rating scales and questionnaires have been developed to assess the prevalence and intensity of all the possible aspects of the psychotherapy process and outcomes [1]. The measurement of processes and outcomes has been one of the most vexing tasks in this area of research because hundreds of process and outcome measures have been created, meaning that there lacks a general consensus regarding what measures are best or how they should be applied in research or practice [2]. When selecting an outcome measure, it must be validated for its intended purpose, as a poor measure can provide inaccurate information about the effectiveness of the interventions.

In counseling education, lectures, demonstrations, case studies, role-playing, presentations, discussions, and reflections are commonly used methods of instruction. These methods aid students in their understanding of the philosophy and concepts of psychotherapy, enabling them to execute learned skills in real-life situations and enhance their personal growth. During demonstration and role-playing, students could play different roles (e.g., counselor, clients, group members, and observers) to practice counseling skills and techniques. Students can witness how others run a group and how the client is treated during counseling. Thus, psychotherapy can be heard, seen, and felt in a learning context [3].

Applying the mirror technique in a group counseling session, the client is placed in a safe experimental situation but mirroring also significantly affects group members [4–8]. While students witness the counseling process, including audio and video case recordings and live demonstrations, it is as if they see themselves in a psychological mirror. As outsiders, this psychological distance allows students to undertake more realistic appraisals of themselves and others. Students reported that they experienced a certain degree of healing effects, the so-called mirror effects [7, 9]. Although mirror effects have healing elements, it is questionable whether mirror effects always lead to positive outcomes.

However, different from the client, in counseling education, people in the group are not intentionally chosen to deal with, or even address, their problems, even though they could have experienced a similar life event to the client. They cannot engage to the same extent as the client, which could cause an emotional outburst but without that person going through catharsis or any therapeutic effects. Triggering the negative mirror effect could exist and is unhelpful. If other people continually escape from their problems, they will not take the initiative to deal with them. It is worthwhile to develop a scale to measure mirror effects to avoid causing harm in counseling education. Given these observations and my intention to develop and validate the Mirror Effects Inventory (MEI), in the first study, a theme analysis was used to identify three categories of mirror effects and further develop the MEI. In the second study, I conducted a principal component analysis (PCA) of the correlations of the MEI.

### Mirroring in counseling education

Mirror, or mirroring, is one of the most powerful therapeutic techniques used in group counseling to enable individuals to reveal their true selves; it was developed by J. L. and Zerka Moreno [10]. In the mirror technique, a person demonstrates that they have understood and perceived what the client conveyed. This person reflects on the client what they have witnessed through action, allowing the clients to feel seen and understood to some extent. The client recognizes themselves in this psychological mirror [11].

Mirroring is rooted in the human mind and prepares us to know the unknown. The two unknowns are: (1) people cannot see their own faces, and (2) people cannot see the other unconscious, on which they depend for their welfare and safety [12–14]. Mirroring entails an emotional association between the inner and outer worlds as well as between subjective and objective realities. It represents the self or the other or complexity and interconnectedness. Mirroring provides us with a bridge that not only connects private and social dimensions but also over the elusiveness inherent in the overture to know oneself and the other. Mirroring encourages clients to observe their behaviors and responses in their interactions with others with greater objective awareness and insight [15]. Clients can undertake some reflection on the input of others and then reenact the scene by themselves after observing their own and others’ responses.

Through mirroring, clients could recognize their limitations at that moment and then accept them gradually. Currently, they could suggest other alternatives at the present moment. Fine redefined mirroring as a model [16]. In some cases, modeling frees the blocked client by permitting them to express thoughts that they have held back until someone else expressed them or by teaching them emotional expressiveness, interpersonal communication, and professional skills or searching for alternatives to create a coping strategy [16]. In a new situation, clients have an opportunity to correct and modify the things they have done previously. They could also learn and develop new problem-solving skills to deal with their future.

In conclusion, there are two functions of the mirror technique: (1) the existential function inspires clients to think objectively about what they are showing subjectively [17]; and (2) the triadic and systemic function sets dyadic confrontations within the context of triangulation to free up deeper feelings and re-evaluate the dynamics of a given situation [18].

The client could have the chance to enact their life without causing trauma, getting hurt, or leaving a scar on their inner heart through mirroring. Mirroring also creates a setting in which members of a group can place themselves in a separate space, watching others speak, and act as if they are doing it. The great impact of mirroring is that it not only influences participants but also arouses their emotions and thoughts (i.e., the observers/students in the classroom, group members, counselors, and teachers) merely witnessing the counseling process. In counseling education, everyone has the “key” and the means to dispute resolution. Each member consciously and unconsciously reflects the inner truths and realities of the other [9]. Within this process of reciprocal reflection, group members can be stimulated to communicate more effectively [19].

Considering this concern, this study focuses on students’ experiences. Students who witness and participate in each other’s personal stories can experience deep empathic understanding. Additionally, it could arouse their own deep emotions toward a given situation or past experience and, as a result, generate a general, positive, or negative effect, termed a mirror effect [7, 9].

### Delineating the mirror effects

The literature [5, 10, 20] supporting the effect of mirroring is noteworthy. Ho categorized and defined three types of mirror effects [9]. A “general mirror effect” is defined as observing a situation in which people develop an empathic understanding or reflect upon their life experiences as they recall events from a certain period. A “positive mirror effect” describes observing a situation in which people learn from others, develop an action understanding, and can execute their skills or knowledge automatically. A “negative mirror effect” is observing a situation in which people engage only in negative emotional outbursts and cannot develop any action understanding, instead indulging in problematic situations and imagery.

One concern relates to the fact that although counseling demonstrations are widely used in the field of education, few researchers have studied the phenomenon, and many have focused only on its therapeutic factors [21]. Another concern is that although mirror reactions describe the type of mirroring [19], the process of mirroring and the therapeutic factors are only addressed from a psychoanalytic perspective; the interpersonal perspective of this mirror effect has not been mentioned.

Therefore, this research seeks to expand the existing base of psychological knowledge concerning mirroring. Although the mirror effects could automatically heal, they could also arouse different levels of emotion in participants. The impact of this arousal of emotion on participants is still uncertain, with most research focused mainly on the impact of group counseling on the client. Consequently, the effects of mirroring remain unclear. The participant responses (i.e., the observers/students in the classroom, group members, and counselors) provide valuable insight for research into counseling education and therapy and its impact on participants and are highly worthy of exploration. This study develops and validates a scale examining the “student experience of affect” in a counseling process, or the so-called mirror effect, which is the emergence of personal experience using PCA.

### Research design

This study arose out of the mirror effects seen in experiential learning from an interpersonal perspective and how this effect can be generated in the field of education in helping professions, especially in counseling. However, the use of experiential learning in counseling education is underdeveloped and underexplored in Hong Kong, and one of the problems of this research was finding appropriate educational institutes in Hong Kong from which to collect data. In addition, counseling must be treated confidentially. The above limitations made it impossible to collect data from other local organizations.

Eventually, the sample of students was chosen by means of purposive sampling from the students at the City University of Hong Kong, where counseling education, conducted by the Professor Lo Tit Wing, adopted experiential learning as a teaching approach [3]. Students from the field of counseling could have sufficient self-awareness to eliminate possible side-effects (e.g., learn from the problematic situation, handle negative emotional states appropriately). Considering ethical issues would reduce or even avoid any harmful consequences and enable students to provide the greatest possible benefit to their clients in future practice.

**Fig. 1:**
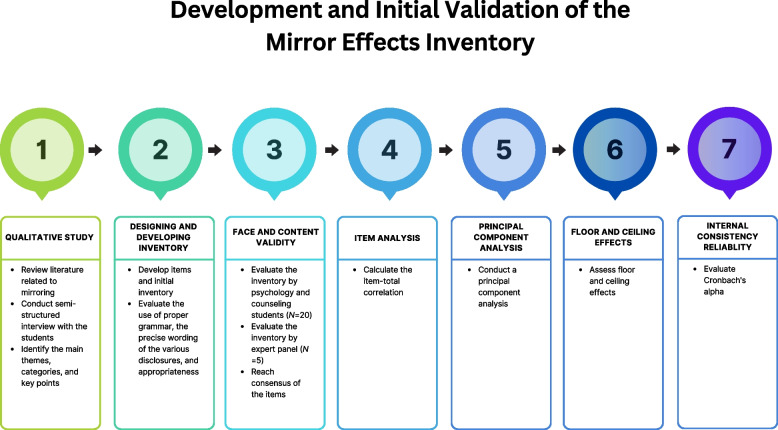
Stages of development of the mirror effects inventory

This study was approved by the College Research Ethics Sub-Committee of the City University of Hong Kong. In the first qualitative study, all former students from a counseling course were invited via email and/or telephone. In the second quantitative study, all current students voluntarily participated in this pre-test and post-test study via class invitation without participating of teaching staff. Their identities were kept anonymous to ensure privacy and confidentiality [22]. Without specific criteria, the selection was not random. Based on purposive sampling, this non-probability sampling has limitations regarding generalization and was not intended to be representative of a population. Figure 1 illustrated the seven stages of development and initial validation of the MEI.

To protect and maximize the benefit to the students, the primary qualitative and quantitative data were kept strictly confidential and were not disclosed to the teaching staff before the period of grading announcement. The analysis only showed the consolidated results, without any information that could identify any person individually.

## Methods

This study used a mixed methods design to develop a scale to measure “student experience of affect” in the counseling education process. First, the qualitative study aimed to identify the main themes of general, positive, and negative mirror effects. Second, the quantitative study aimed to validate the scale. Psychometric tests were performed on the data collected from MEI to examine item performance, validity evidence, and reliability estimates and psychometric properties to evaluate the overall quality and pinpoint areas that could require revision or improvement. This study was not preregistered.

### Study one: qualitative study

The mirror effect is an abstract concept. Studies have rarely examined the psychological aspects of the mirror effect; therefore, this study consisted of two parts. In the first study, to collect empirical data and map the main themes of the experiential learning approach and mirror effects, students were surveyed via a telephone semi-structured interview. Based on the interview data, a subjective perceived scale of mirror effects was constructed. This preliminary analysis aided in developing a theoretical concept and the construction of a questionnaire for the second study.

#### Participants

Respondents were former students who completed a group counseling course at the City University of Hong Kong. Twenty-four of the 30 telephone invitees were successfully contacted and voluntarily participated in a telephone interview.

In this study, 41.7% of respondents had studied at the bachelor’s degree level, and 58.3% had studied at the master’s degree level. Of these, 79.2% were required to provide counseling services in their workplace, 70.8% were working in social service centers, and 12.5% were working in the education sector. Respondents had differing levels of counseling experience, with 29.2% having individual and group counseling experience exceeding nine years. Although the sample was insufficient for generalization, the advantage of this study was the variety of respondents’ experiences and their service nature, which provided extensive insight while allowing them to use the lessons learned in their workplaces. The information gained (Table 1) provided diverse interpretations of mirror effects regarding respondents’ educational attainment and working experiences, as well as an opportunity for them to use the knowledge gained from their counseling courses and bachelor’s and master’s programs.

**Table 1:** Summary of sample characteristics in study one (*N* = 24)

	***n***	**%**
**Sex**		
Male	4	16.7
Female	20	83.3
**Educational** a**ttainment**		
Bachelor	10	41.7
Master	14	58.3
**Nationality**		
Chinese	24	100
**Occupation**		
Administration	3	12.5
Social service	17	70.8
Education (i.e., teaching and providing counseling services)	3	12.5
Other (i.e., nurse)	1	4.2
**Counseling** e**xperience**		
0–2 years	6	25
3–5 years	8	33.3
6–8 years	3	12.5
≥ 9 years	7	29.2
**Provide counseling services in workplace**		
Required	19	79.2
Not required	5	20.8

#### Procedure

A consent form with a declaration was sent to each respondent prior to the date of the interview, explaining the purpose, procedures, benefits, and any potential liabilities inherent in participating in a half-hour audiotaped telephone interview.

The interview questions (see Additional file 1) were held constant for each respondent regarding wording and order. A semi-structured format was used to elicit richer and more elaborate content because it is more detailed than a structured format. The respondents were interviewed to document (a) feelings, thoughts, behaviors, attitudes, perceptions, and experiences toward mirror effects; and (b) insight, personal growth, and integration on cognitive, affective, and behavioral levels after participating in counseling education.

#### Data analysis

Through qualitative analysis, the first step was to confirm the existence of mirror effects in counseling education, and the second step was to investigate students’ experiences of mirror effects. Qualitative verbatim quotes from the interviewees were integrated to explore and reconfirm the existence of mirror effects.

The second component was a thematic analysis of each respondent included in the qualitative study sample in the form of one report for all respondents. The themes of the dataset were used to describe and categorize the phenomenon of mirror effects in the readiness for analysis. To establish meaningful patterns, the data were analyzed through a six-phase coding process: familiarization with the data, generating initial codes, searching for themes, reviewing themes, defining and naming themes, and producing the final report [23].

After conducting all interviews, the audio recordings were transcribed by four trained research assistants before being reviewed and checked by the researcher. The researcher read and re-read the transcriptions, noted initial ideas related to mirror effects, coded the transcripts, and categorized the data on the perceived condition of mirror effects and other related variables. The transcripts were read, reviewed, and checked by a doctoral-level rater with an academic writing background who evaluated the coding and classification scheme in this study and checked the transcriptions for accuracy to avoid bias and misinterpretation [24].

Using an open coding method, patterns and themes were categorized based on the data. After the data from all respondents were coded by the researcher to identify and categorize specific observable actions or characteristics, similarities and differences between respondents were listed and re-coded to ascertain emergent categories in exploratory form. This data reanalysis involved classifying, categorizing, and identifying themes and patterns for each student from the interview and then dividing the codes into a logical order to create a final coding scheme. Recurring themes and patterns, and the interrelationships between these themes and patterns, were established from the data.

The rater was used to validate the codes and themes independently as well as to review the analysis and interpretation of the themes and patterns. To reach a consensus, a final report was produced.

## Results

Concerning perceptions of mirror effects, 159 statements were extracted. Regarding the mirror effects, 113 statements were identified. Further, 92 statements were connected to participants’ experiences after participating in counseling education. Several new themes previously not documented in the literature were found, including retrieving past memories, reviewing past events, a reflection of self, and three types of mirror effects (general, positive, and negative). These statements were then categorized into 37 key themes (Table 2).

**Table 2:** Main themes and key points categorized from the former students’ interview transcripts (*N* = 24)

Main themes	Categories	Key points coded from interview data
**General mirror effect**	Problem-solving	1. Learn to handle significant events
		2. Feel in control of my problem
		3. Execute the learned knowledge and/or skill to deal with significant events
		4. Learn to solve interpersonal conflict
	Insight	5. Gain insight
**Positive mirror effect**	Support	6. Gain support from my classmates
		7. Gain support from my teachers
	Interpersonal skills	8. Discern the use of interpersonal skills
	Ventilation	9. Feel being ventilated
		10. Express suppressed emotion
	New thinking	11. Modify unrealistic expectations
		12. Develop a positive attitude to deal with difficulty
		13. Regain vigor
		14. Inspire new thinking
		15. Find a clear goal
		16. Gain new perspective
	Re-evaluation the past	17. Recall past events
		18. Associate similar experiences
		19. Re-evaluate the pain of stressful events
	Self-understanding	20. Develop self-reflection
		21. Develop self-criticism
		22. Evaluate myself
		23. Gain self-knowledge
**Negative mirror effect**	Poor problem-solving skills	24. Use negative approach to handle problem
		25. Avoid solving the problem
		26. Escape from the situation
		27. Indulge in problematic situation and/or imagery
		28. Indulge in negative feelings
	Blaming	29. Blame other people
		30. Blame myself
	Low self-confidence	31. Have low self-confidence
		32. Feel of low courage
	Negative emotions	33. Feel fear
		34. Feel sorrow
		35. Feel regret
		36. Feel shame
		37. Feel rejected

The 37 key themes related to mirror effects were integrated into 11 categories. The main themes of mirror effects emerged after coding and categorizing them into three subscales: general, positive, and negative. These qualitative data were then used to develop a questionnaire for Study Two.

### Study two: quantitative study

#### Hypotheses

After performing the PCA on mirror effects, validation of the MEI was performed by testing hypotheses based on correlations between the three types of mirror effects and sex, age differences, educational attainment, and occupation between each type of mirror effects. It was hypothesized that the general mirror effect is positively correlated with the positive mirror effect (H_1_) and negatively correlated with the negative mirror effect (H_2_), and the positive mirror effect is negatively correlated with the negative mirror effect (H_3_). Life experiences and education serve as catalysts for the development of problem-solving skills, the acquisition of insights, and strengthening interpersonal skills. Specifically, it was predicted that general mirror effect is positively correlated age (H_4_) and educational attainment (H_5_), positive mirror effect is positively correlated age (H_6_) and educational attainment (H_7_), and negative mirror effect is negatively correlated age (H_8_) and educational attainment (H_9_).

#### Participants

A total of 221 participants were part-time university students enrolled in undergraduate and graduate programs, studying counseling courses at the City University of Hong Kong, and completed the survey with most participants aged 21–30 years of age (58.5%). Over half were women (67.7%), most participants held employment in social services (58.9%), and all had tertiary education (100%). Participants’ demographic characteristics are presented in Table 3.

**Table 3:** Demographic characteristics of participants (*N* = 221)

	***n***	**%**
**Sex**		
Male	63	32.3
Female	132	67.7
**Age (years)**		
16–20	1	0.5
21–25	53	27.2
26–30	61	31.3
31–35	33	16.4
36–40	21	10.8
41–45	17	8.7
46–50	6	3.1
51–55	4	2
**Educational attainment**		
Tertiary education (e.g., associate degree, higher diploma)	52	26.4
University	92	46.7
Master’s	48	24.4
Ph.D	5	2.5
**Occupation**		
Art/entertainment	2	1.0
Administration	15	7.6
Building/techniques	1	0.5
Business management	3	1.5
Counseling	2	1.0
Education	13	6.6
Information technology	1	.5
Hospital/medical	7	3.6
Human resources	7	3.6
Marketing	7	1.5
Social services	116	58.9
Full-time student	21	10.7
Other	6	3.0

#### Measures

The MEI, a self-constructive inventory, examines the occurrence of mirror effects after participation in counseling education using three subscales: general, positive, and negative. The inventory comprises 37 items rated on a five-point Likert scale (1 = *strongly disagree;* 5 = *strongly agree*). Higher scores reflect a greater presence of mirror effects.

#### Procedure

The study had a descriptive quantitative cross-sectional design, and data collection for the survey was completed via self-administered questionnaires (see Additional file 2) in paper-and-pencil format during academic lectures. Informed consent was obtained to confirm that the individual understood the information provided and agreed to participate.

#### Data analyses

Descriptive statistics were calculated for the demographic information. The internal consistency reliability of the scale was explored by computing Cronbach’s alpha of the MEI scale and the positive, negative, and general subscales. One problem with the questionnaire relates to the fact that there were five cases of missing MEI data and missing values per case is one. In general, the approaches for handling missing data in a dataset are to delete samples with missing data values, impute the value of the missing data, or remove a variable. It is relatively common to have missing data in almost all research, and it can significantly affect the conclusions that can be drawn from the data [25].

Kline proposed that a sample size greater than 200 is large [26]. Tabachnick and Fidell suggested that for 5% or less missing points at random in large datasets, there is little risk of serious problems, and any method to handle missing data is acceptable [27]. Davey and Savla stated that if missing values are reasonably random and the extent of the problem is not large, the mean substitution method can be used to calculate a mean based on the valid cases and to replace the missing values [28]. Another method involves directly applying methods that are unaffected by missing values. The missing values in the current MEI dataset were unaffected and direct analysis was used. Additionally, non-response to participant characteristics is common in research, and several participants did not provide demographic information. Missing demographic information could occur for several reasons, including refusal to provide personal information or an inaccurate understanding of the requested information [29]. Notably, omitting participants with missing data often leads to reduced statistical power, biased estimates, and consequently inaccurate inferences [29]. Therefore, as the missing information pertained to demographic information only, apart from five missing MEI data points as discussed previously, a decision was made to include all the MEI data as inferences relative to the reliability, and factor components of the MEI would still be accurate and reliable.

The second problem was that the questionnaire data were collected in the first lecture, within the add/drop period, and the last lecture of the 13-week course; therefore, the sample sizes were unequal. Twenty-two students did not complete the pre-test questionnaire. However, students were asked to record the identity number of the pre-test (*n* = 198) and write it on the post-test (*n*= 221). Only 198 pre-test and post-test questionnaires were successfully matched. The data are entered into the same row in separate columns. Some demographic characteristics were not collected during the post-test stage. SPSS 28 (IBM, Armonk, NY, USA) and AMOS 29 (IBM, Armonk, NY, USA) automatically use the right formula for data analysis. MEI examines mirror effects following counseling education. Apart from this, there is evidence that some questions positioned later in a lengthy questionnaire additionally suffer from a lower quality of responses [30]. With these concerns, MEI was gathered at post-test only. Therefore, the data of this study were based only on the post-test.

As mentioned in Fig. 1., the process of this study had seven stages. After item generation (first stage), comprehensibility of the items was evaluated (second stage). The psychometric properties of MEI were evaluated in a current and logical direction, including face and content validity (third stage), item analysis (fourth stage), PCA (fifth stage), floor and ceiling effect (sixth stage), and internal consistency reliability (seventh stage) [26].

### Data availability statement

The datasets presented in this study can be found in online repositories. The names of the repository/repositories and accession number(s) can be found at: https://osf.io/teka7/files/osfstorage/63996053e4687409c79b4b1c.

## Results

### Face validity

Face validity refers to how an instrument appears to measure what the researcher intended it to measure, at face value. Researchers must rely on judgment rather than empirical evidence when determining face validity. Face validity was evaluated with qualitative and quantitative approaches. In the qualitative approach, the inventory was sent to 20 psychology and counseling undergraduate and graduate students who were asked to assess the inventory in terms of difficulty, relevancy, and ambiguity. All items were understandable to participants.

Students were asked to assess the items in terms of suitability using a five-point Likert scale (1 = *it is not suitable*; 5 = *it is completely suitable*). The impact score was calculated from the formula of frequency (%) × suitability. A score of 1.5 was considered acceptable [31]. The impact scores for all items were obtained with a rating of > 1.5, indicating the items had acceptable face validity. Thirty-two items were accepted, and five items changed in wordage.

### Content validity

Content validity refers to the items representing the content the inventory is designed to measure. Content validity has been established through subjective and qualitative processes, as well as through quantitative processes [32]. To examine the content validity of the MEI, this study followed the procedure established by Cottrell and McKenzie [33]. The content validity index (CVI) and the content validity ratio (CVR) were determined after item generation and item selection based on literature review and qualitative analysis, and items were modified by an expert panel (*N* = 5). The CVI was rated by the experts specializing in research, education, and counseling psychology (the researcher, two raters, and two teaching staff) on a four-point Likert scale (1 = *strongly not relevant;* 4 = *highly relevant*) and then calculate using the averaging approach. The formula for CVR = (N_e_ − N_t_ /2)/(N_t_ /2), in which N_e_ denotes the number of experts indicating “3 = *quite relevant*” and N_t_ denotes total number of experts. The formula for CVI = CVR = (N_e_/N_t_), in which N_e_ denotes the number of experts indicating “3 = *quite relevant*” and N_t_is total number of experts. Lawshe proposed that the minimum acceptable cut‑off for CVR is 0.99 when five experts are used [34].

The experts were asked about some of the qualitative characteristics of items, such as compliance to principles of grammar and wording. Finally, they agreed that 30 items should be accepted without any changes and seven items should be reworded. After consensus was reached, the inventory was rerated by each expert individually. The content validity was good with item-level CVI was 1, indicating all items had acceptable content validity and values of CVR were 1, indicating each item was relevant [34].

### Item analysis

An analysis was conducted to identify possible problems of items by computing the corrected item-total correlations. The correlation coefficient between items less than 0.3 and greater than 0.9 were omitted [31]. At the stage of item analysis, no items were deleted.

### Correlations among factors

Intercorrelations among factors are presented in Table 4 and indicate that discriminant validity is achieved when the correlations between factors do not exceed 0.70. The *general mirror effect* had weak, positive, and significant correlations with the positive mirror effect (*r* = 0.17, *n* = 221, *p* < 0.05; supported H_1_), and a moderate, negative, and significant correlation with the negative mirror effect (*r* = -0.47, *n* = 220, *p* < 0.001; supported H_2_). The *negative mirror effect* had only a weak, positive, and significant correlation with the positive mirror effect (*r* = 0.14, *n* = 219, *p* < 0.05; rejected H_3_). Increasing in the general mirror effect might increase positive mirror effect and decrease negative mirror effect; however, increasing in the positive mirror effect might increase negative mirror effect, thereby partially supporting the hypotheses.

**Table 4:** Correlation matrix among the mirror effects inventory factors (N = 221)

Factor	1	2	3	4	5	6	7
1. General mirror effect	-						
2. Positive mirror effect	.17*	-					
3. Negative mirror effect	-.47***	.14*	-				
4. Sex	.02	.13	.07	-			
5. Age	.19**	-.04	-.06	-.11	-		
6. Educational attainment	-.37***	.01	.19**	-.04	.16*	-	
7. Occupation	-.07	.05	.16*	.06	-.16*	-.15*	-

^*^p < .05

^**^p < .01

^***^p < .001

Additionally, the general mirror effect had weak, positive, and significant correlations with age (*r* = 0.19, *n* = 195, *p* < 0.01; supported H_4_), and a weak, negative, and significant correlation with the educational attainment (*r* = -0.37, *n* = 197, *p* < 0.001; rejected H_5_). However, there is no correlation between *positive mirror effect* and age (*r* = -0.04, *n* = 194, *p* = 0.56) and educational attainment (*r* = 0.01, *n* = 196, *p* = 0.94; rejected H_6_ and H_7_). The *negative mirror effect* had no correlation with age (*r* = -0.06, *n* = 194, *p* = 0.38; rejected H_8_) and weak, positive, and significant correlations with educational attainment (*r* = 0.19, *n* = 195, *p* < 0.01; rejected H_9_) and occupation (*r* = 0.16, *n* = 195, *p* < 0.05). Age had weak, positive, and significant correlations with educational attainment (*r* = 0.16, *n* = 195, *p* < 0.05), and weak, negative, and significant correlations with occupation (*r* = -0.16, *n* = 195, *p* < 0.05). Educational attainment had weak, negative, and significant correlations with occupation (*r* = -0.15, *n* = 197, *p* < 0.05). Increasing in age might increase general mirror effect. High educational attainment might decrease general mirror effect and increase negative mirror effect.

### PCA

PCA is a method to identify the maximum variance among a set of items by creating linear functions of those items, which required to explain the total variance observed in the correlation matrix for an item set [35–37]. PCA is used to identify underlying dimensions of response patterns that best predicts the set of questionnaire responses. It is widely accepted as an appropriate way to develop questions that are valid and reliable [35, 38, 39]. In the process of measure development, PCA is most helpful when used to describe the strengths and weaknesses of the measure, including its content validity and structural validity/factorial validity [40]. PCA is more appropriate when refining a measure (e.g., identifying dimensions that need more items, reducing the total number of items for future administrations of the measure) [41].

PCA was conducted on the MEI scale items with orthogonal (varimax) rotation for the factor structure*.*PCA is a dimensionality reduction technique that reduces variables to principal components and retains as much variation as possible within an original dataset [42]. Rotation in PCA assists in understanding the component loadings [43].

Determination of the number of factors extracted from a PCA to be retained, decision rules that can be applied after an analysis is complete are Kaiser’s criterion, scree test, and parallel analysis. According to Kaiser’s criterion, all factors with eigenvalues greater than one are retained. However, the criterion has been criticized because of resulting in the retention of too many factors [44]. Seven factors were greater than one. Cattell’s scree test was then employed, and the curve begins to tail off after four factors. Cattell recommends retaining all factors above the elbow, or break in the plot, since these factors contribute the most to the explaining variance in the set of data [45]. The scree plot showed inflections, justifying the retention of the three factors. The sample size was less than 300. A parallel analysis was conducted by using Monte Carlo PCA to generate sets of random data to be compared with eigenvalue obtained in SPSS. Horn's parallel analysis, one of the most accurate approaches, which involves comparing the size of eigenvalues that exceed the corresponding values from the random dataset are retained [46, 47]. The result of parallel analysis for MEI demonstrated that Component/Factor 1, 2, and 3 are retained and have strong load values that exceed the corresponding criterion values for a randomly generated data matrix of the same size (27 items × 221 respondents). The three components/factors contain positive loadings. Hence, results from Kaiser’s criterion, Scree test, and parallel analysis explained three components were better to retain.

A three-factor structure was obtained based on the PCA, and 37 items were loaded on each component with loadings ≥ 0.3 [35]. The Kaiser–Meyer–Olkin (KMO) measure verified the sampling adequacy for the analysis, KMO = 0.90 (“marvelous,” according to Kaiser) [48, 49], and Bartlett’s test of sphericity was significant (χ^2^ (666) = 5695.560, *p* < 0.001). The diagonals of the anti-image correlation matrix were all above 0.5, and all commonalities were above 0.3, confirming that each item shared a common variance with the other items. All factors together explained 53.82% of the total variance, and the three components were labeled (1) negative mirror effect, (2) positive mirror effect, and (3) general mirror effect. Table 5 lists the factor loadings after rotation.

**Table 5:** Factor loadings based on a Principal Component Analysis with Oblique Rotation for thirty-seven items of the mirror effects inventory (*N* = 221)

Item	1	2	3
(G3) I learn to handle significant events			**.80**
(G2) I execute the learned knowledge and/or skill to deal with significant events			**.78**
(G5) I gain insight			**.77**
(G1) I feel in control of my problem			**.75**
(G4) I learn to solve interpersonal conflict			**.74**
(P11) I regain vigor		**.73**	
(P13) I find a clear goal		**.68**	
(P10) I gain support from my teachers		**.66**	
(P8) I express suppressed emotion		**.64**	
(P14) I gain new perspective		**.63**	
(P12) I inspire new thinking		**.58**	.38
(P17) I evaluate myself		**.57**	
(P5) I re-evaluate the pain of stressful events		**.56**	
(P7) I feel being ventilated		**.56**	
(P2) I develop a positive attitude to deal with difficulty		**.55**	
(P9) I gain support from my classmates		**.55**	
(P4) I associate similar experiences		**.55**	
(P15) I develop self-reflection		**.55**	
(P6) I modify unrealistic expectations		**.54**	
(P16) I develop self-criticism		**.54**	
(P18) I gain self-knowledge		**.53**	
(P1) I discern the use of interpersonal skills		**.52**	
(P3) I recall past events	.31	**.36**	
(N8) I feel fear	**.88**		
(N5) I feel of low courage	**.87**		
(N12) I feel rejected	**.86**		
(N11) I feel shame	**.84**		
(N14) I blame myself	**.83**		
(N9) I feel sorrow	**.80**		
(N7) I escape from the situation	**.80**		
(N6) I avoid solving the problem	**.79**		
(N4) I have low self-confidence	**.79**		
(N10) I feel regret	**.78**		
(N3) I indulge in negative feelings	**.77**		
(N13) I blame other people	**.74**		
(N2) I indulge in problematic situation and/or imagery	**.69**		
(N1) I use negative approach to handle problem	**.41**		-.57
Eigenvalues	10.60	6.52	2.80
Percentage of total variance	28.65	17.62	7.55
Number of test measures	14	18	5

Extraction method: Principal component analysis (PCA), rotation method oblimin with Kaiser nor malization. Factor loadings below .3 are not displayed

The first component/factor, negative mirror effect, contained 14 items and accounted for 28.65% of the variance. Items represented negative responses (e.g., negative emotional outbursts) that students experience after participating in counseling education. Sample items include “I feel fear” and “I avoid solving the problem.”

The second component, positive mirror effect, contained 18-items and accounted for 17.62% of the variance. Items represented positive responses (e.g., develop an action understanding) that students experience after participating in counseling education. Sample items include “I gain new perspective” and “I re-evaluate the pain of stressful events.”

The third component, general mirror effect, contained five items and accounted for 7.55% of the variance. Items represented general responses (e.g., reflect upon their life experience) that students experience after participating in counseling education. Sample items include “I learn to handle significant events” and “I gain insight.”

### Floor or ceiling effects

Floor and ceiling effects are considered as optimal if less than 15% of respondents achieved the lowest or highest possible score [50]. If floor or ceiling effects are present, it is likely that extreme items are missing in the lower or upper end of the scale, indicating limited content validity. The floor and ceiling effects were analyzed, taking as acceptable effects those that were < 15% (Table 6).

**Table 6:** Floor and ceiling effects on the three factors of MEI

Variables	N	Minimum	Maximum	Floor effect	Ceiling effect
1. General mirror effect	221	7	25	14 (6.33)	3 (1.36)
2. Positive mirror effect	220	32	89	5 (2.27)	3 (1.36)
3. Negative mirror effect	219	14	68	0 (0)	1 (.46)

Discrete values as number (percentage)

The skewness of the general mirror effect was -1.23, indicating that the distribution was left-skewed (highly negative skewed). The skewness of the positive mirror effect was -0.81, indicating that the distribution was left-skewed (moderately negative skewed). The skewness of the negative mirror effect was 0.22, indicating that the distribution was fairly symmetrical. The kurtosis of the general and negative mirror effects was 1.21 and -0.34, indicating that the distribution was normal. The kurtosis of the positive mirror effect was 3.46, indicating that the distribution was more heavy-tailed compared to the normal distribution (Table 7).

**Table 7:** Skewness and Kurtosis for Normality Test

Variables	Skewness		Kurtosis	
	**Statistic**	**Std. Error**	**Statistic**	**Std. Error**
1. General mirror effect	-1.23	.16	1.21	.33
2. Positive mirror effect	-.81	.16	3.46	.33
3. Negative mirror effect	.22	.16	-.34	.33

### Internal consistency and reliability

Internal consistency refers to a measure of the extent that items in a questionnaire (sub)scale are correlated (homogeneous). Cronbach’s alpha was used to measure the internal consistency and reliability of the items. George and Mallery proposed that 0.7 level of higher of Cronbach’s alpha is acceptable [48, 50, 51]. The MEI was reliable (Cronbach’s alpha = 0.88), with most the corrected item-total correlations being ≥ 0.4 (Table 4). Cronbach’s alpha for the subscales was as follows: general mirror effect, *α* = 0.90; positive mirror effect, *α* = 0.88; and negative mirror effect, *α* = 0.96. The descriptive statistics of the total and individual items for the subscales are presented in Table 8.

**Table 8:** Item-total correlation for the mirror effects inventory (*N* = 221)

Item	Pearson’s *r*	Alpha^a^	Mean (SD)
**Mirror Effects Inventory total**		.88	121.50 (13.38)
**General mirror effect**		.90	18.16 (3.64)
(G1) I feel in control of my problem	.77	.87	3.61 (0.76)
(G2) I execute the learned knowledge and/or skill to deal with significant events	.82	.86	3.70 (0.81)
(G3) I learn to handle significant events	.84	.85	3.67 (0.85)
(G4) I learn to solve interpersonal conflict	.67	.89	3.52 (0.80)
(G5) I gain insight	.68	.90	3.64(1.09)
**Positive mirror effect**		.88	68.05 (7.11)
(P1) I discern the use of interpersonal skills	.45	.87	3.80 (0.58)
(P2) I develop a positive attitude to deal with difficulty	.50	.87	3.92 (0.55)
(P3) I recall past events	.35	.88	3.72 (0.86)
(P4) I associate similar experiences	.54	.87	3.80 (0.80)
(P5) I re-evaluate the pain of stressful events	.53	.87	3.82 (0.76)
(P6) I modify unrealistic expectations	.49	.87	3.64 (0.78)
(P7) I feel being ventilated	.47	.87	3.39 (0.92)
(P8) I express suppressed emotion	.57	.87	3.46 (0.84)
(P9) I gain support from my classmates	.45	.87	3.63 (0.69)
(P10) I gain support from my teachers	.54	.87	3.62 (0.73)
(P11) I regain vigor	.63	.87	3.75 (0.70)
(P12) I inspire new thinking	.52	.87	4.00 (0.57)
(P13) I find a clear goal	.61	.87	3.73 (0.61)
(P14) I gain new perspective	.54	.87	3.92 (0.57)
(P15) I develop self-reflection	.48	.87	4.12 (0.50)
(P16) I develop self-criticism	.49	.87	3.86 (0.67)
(P17) I evaluate myself	.52	.87	3.91 (0.61)
(P18) I gain self-knowledge	.46	.87	3.95 (0.57)
**Negative mirror effect**		.96	35.30 (11.06)
(N1) I use negative approach to handle problem	.51	.96	2.61 (1.06)
(N2) I indulge in problematic situation and/or imagery	.70	.95	2.70 (1.01)
(N3) I indulge in negative feelings	.68	.95	2.32 (0.90)
(N4) I have low self-confidence	.81	.95	2.56 (0.97)
(N5) I feel of low courage	.85	.95	2.47 (0.96)
(N6) I avoid solving the problem	.77	.95	2.62 (0.98)
(N7) I escape from the situation	.76	.95	2.53 (0.92)
(N8) I feel fear	.86	.95	2.46 (0.97)
(N9) I feel sorrow	.77	.95	2.68 (1.11)
(N10) I feel regret	.75	.95	2.78 (1.10)
(N11) I feel shame	.86	.95	2.47 (1.03)
(N12) I feel rejected	.87	.95	2.34 (0.93)
(N13) I blame other people	.73	.95	2.24 (0.93)
(N14) I blame myself	.75	.95	2.52 (1.02)

^a^Indicates internal consistency of remaining items if the elected item were deleted from the total score

## Discussion

This study developed a novel self-report measure that examines mirror effects and evaluates the psychometric properties of the scale. Additionally, it aimed to share the phenomena observed in counseling courses and researchers’ experiences on mirror effects to provide other researchers with opportunities to expand the existing literature on psychological knowledge concerning mirroring. The MEI scale showed a high degree of total internal consistency reliability and reasonable content validity, and the results supported a three-factor model of the MEI.

In group counseling, mirroring can foster self-reflection and self-awareness by allowing a person to step back and observe themselves as others would view them [7, 52, 53]. When a person engages in this form of experiential learning, it is observed through a psychological mirror that generates general and positive mirror effects [7, 54]. A correlation was observed between the three types of mirror effects. The result implies that a general mirror effect could benefit both positive and negative mirror effects. Learning problem-solving skills or gaining insights, for example, might increase positive mirror effects and decrease negative mirror effects; however, increasing positive mirror effects might increase negative mirror effects. Thus, it is possible for positive and negative mirror effects to coexist concurrently. For example, people could have negative emotions and self-blaming while re-evaluating their past. People could give themselves the best chance to live a balanced and meaningful life. Every one of us experience ups and downs in different stages of life. It encompasses all the events interactions and challenges we have encountered throughout our life including positive and negative experiences. Life experience shapes who we are and influences our perspectives beliefs, values, attitudes, and behaviors. Lessons learned are the knowledge gained through analyzing the successes and failures occurring in one’s own life. The most effective life lessons are learned through experience. This aligns with the principle of experiential learning.

The avoidance of experiential learning and mirroring could inhibit a counselor from successfully relating to their clients, as they could be unable to engage effectively with clients who present with similar lived experiences [55]. This could create a therapeutic environment in which unanticipated emotional outbursts could materialize, which could impede cathartic or therapeutic effects, and the triggering of a negative mirror effect could emerge and be detrimental to the therapeutic process [7, 56, 57]. Moreover, if people continuously avoid their problems, their motivation and initiative to deal with them will remain absent [58].

Within counseling education, the importance of mirror effects and associated therapeutic effects is related to the successful integration of theory and practice [7, 59]. Additionally, mirror effects are valuable for educators and helping professionals, as students can gain new perspectives and experience emotional and active cognitive processing, which could result in positive therapeutic effects, and enhance future performance [7, 60] As such, developing and examining a measure that examines mirror effects seems warranted to avoid unnecessary harm in counseling education. Additionally, it can encourage students to replace personal maladaptive thoughts with more adaptive ones and help them cope with past experiences and behaviors by processing negative thoughts and emotions in a psychologically safe environment [7, 60].

In addition, the results showed a weak relationship between age and general mirror effect but not positive and negative mirror effects. Increasing in age increased general mirror effects. In this respect, the findings differed from previous study. Artistico et al. reported that younger and middle-aged adults usually outperform older adults when it comes to solving everyday problems [61]. High educational attainment decreased general mirror effects and increased negative mirror effects. This aligned with previous findings showing that an individual’s academic achievement has little impact on their ability to solve problems [62]. Perhaps people with high educational attainment have more comprehensive academic experiences and emotions. These experiences and emotions could have different influences on learning effects; however, these have not been systematically studied [63]. Researchers should explore these phenomena.

Scale development and validation is a rigorous process and a crucial part of the social, behavioral, and health sciences [64]. Scales are developed to capture a latent construct, and several steps are required to ensure that an instrument is both valid and reliable and to measure the intended complex phenomena [64, 65]. Researchers are not uncommon to bypass the exploratory process and direct their efforts toward the confirmatory stage [64]. However, exploratory methods within the initial stage of scale development are recommended because they avoid future problems associated with the scale validity [66].

Before conducting reliability and factorial analysis, exploratory methods should be applied [64, 66]. The first stage of scale development is item development, which involves the identification of a specific domain, item generation, and consideration of content validity [64]. Thematic analysis was conducted in study one to identify, describe, and categorize relevant themes of the mirror effect phenomenon. Item reduction following the thematic analysis resulted in 37 key themes. A PCA was used to examine the dimensionality of the scale and investigate the latent structure of the scale items and their underlying relationships [64]. The analyses confirmed that the MEI successfully discriminated between three mirror effects: positive, general, and negative. Reliability analysis demonstrated that the MEI had excellent internal consistency reliability, *α*= 0.88. Cronbach’s alpha is the most widely used index to test the reliability of a scale and to measure the internal consistency of scale items based on item intercorrelations [48, 64]. An alpha coefficient of 0.70 is considered an acceptable threshold for reliability, validity, and concurrent (criterion) validity [64]. However, to date, no other studies provide measures for assessing mirror effects; therefore, concurrent validity was unable to be examined owing to a lack of a comparable “gold standard” measure.

Additionally, the author was not aware of a similar measure that could be used to test convergent validity. The MEI demonstrated excellent content validity, suggesting that the scale is adequate for assessing mirror effect phenomena and provides evidence of content relevance, representation, and technical quality [48, 64]. Thus, the MEI is an effective measure for assessing mirror effects. This is consistent with previous research [67], whereby the construct validity and reliability of the 14-item Reflection-in-Learning Scale was examined, with research findings supporting the measures construct validity and internal consistency of reflective practices among 196 university students (*α*= 0.86). Further, the findings align with previous research by Lingley-Pottie and McGrath [68], whereby excellent content validity was found during the scale development and validation of the treatment barrier index scale; thus, the scale was considered acceptable for future use in healthcare settings to determine participant perspectives of treatment delivery [69]. The findings suggest that the MEI maintains promise as an outcome measurement appropriate for meeting the distinctive needs of a clinical education, training, and practice.

However, the phenomenon of mirror effects was observed in counseling courses and further informed the MEI. This new measure is considered valid until other studies using the measure and the results of their validity tests are replicated [70]. A confirmatory factor analysis needs to be used in subsequent phases of instrument development to establish construct validity [71].

The representativeness of the sample could be questioned, and comprehensive cross-agency data on the students could be considered for further analysis. Mirror effects could generate in other helping profession training (e.g., nursing, special education teacher training). It is therefore important to conduct research in other disciplines to reveal the deeper meaning of mirror effects. International testing of the MEI would further support its use in a wider population. Psychological research has examined diverse samples to better understand human experiences [72]. Therefore, attempts to establish generalizability across populations require consideration of scale validity and constraints on generalizability across cultural contexts [73]. Lehman et al. suggested that cultural differences in relational schemas influence interpersonal dynamics, and culture influences psychological processes [74]. Moreover, compared to individualistic cultures, collectivistic cultures appear more sensitive to the relational context, with emphasis placed on group benefits, opinions, and harmony [74]. Therefore, it is relevant to explore cultural differences and similarities in mirror effects via the MEI to contribute to understanding interpersonal dynamics and culture.

This study has several limitations. First, the use of convenience samples made it difficult to determine the number of eligible participants unable to participate in the research. Second, the sample consisted only of Chinese participants (*N*= 221), and the scale had to be tested within other culturally diverse samples to support the generalizability of the scale. Cross-cultural validity is a type of construct validity that relates to the degree that scale items can be culturally adapted, and the performance of those items can adequately reflect the performance of the original scale items [75]. Further, cross-cultural validation will provide support for a scale to be considered culturally applicable, meaningful, and equivalent to other cultures [72, 76]. Thus, implementing the MEI in different cultures will support external validity measures.

The current findings have theoretical and practical implications for our understanding of mirror effects and the benefits of experiential learning of mirroring in a group counseling setting. Additionally, this study addressed the issue that to date, no scale measures the mirror effect phenomenon, highlighting the need for developing and validating a relevant scale. Measurements play an integral role in clinical practice and research. Thus, evaluating the quality of a measurement is essential to ensure that the results are accurate and appropriately applied in clinical practice [77]. Psychometric research is concerned with the optimal relationship between psychological constructs and observable outcomes [78]. Therefore, an instrument must demonstrate psychometric soundness, indicating sufficient levels of validity and reliability for ethical use within research [79]. Overall, well-designed scales are the foundation of the scientific understanding of latent constructs, and the MEI could be considered a valid and reliable measure for assessing mirror effects in counseling settings.

The study has several clinical and practical implications. First, the MEI scale could serve as a measurement of the counseling outcomes in both clinical practices and education. Counseling process refers to the characteristics, events, or conditions that occur during and as a result of the interaction between the counselor and client. Process refers to what counselors do with their clients and how clients change over time. Contrastingly, outcomes related to the effects or results of counseling [80]. Previous studies primarily employed process measures (e.g., Client Reactions [81]) and outcome measures (e.g., Beck Depression Inventory [82]) in clinical practice, and only a few addressed process measures (e.g., Counseling Self-Estimate Inventory [83]) and outcome measures (e.g., Client Evaluation of Counseling Inventory [84]) in clinical education. Therefore, the utilization of the MEI as an outcome measure can assist clinicians in developing a deeper more intuitive understanding of their professional competence and support clinicians in their professional development, which can assist clinicians in understanding what aspects of their clinical practice are effective and beneficial for their clients [80]. Additionally, the MEI can enhance greater work outcomes such as job commitment and satisfaction [85].

Second, MEI was developed from an interpersonal perspective to measure “student experience of affect” in the counseling education process. This study identified that the MEI can be utilized as an effective measure in clinical education to develop an understanding of mirror effects that may enhance personal awareness and reduce unnecessary harm through reflective practice. The result of the measurement can be used as a reference to realize their experience of affect tied to some earlier aspect of their life, current learning process, or others’ behaviors. Thus, students should continue to review themselves through learning counseling knowledge and skills as well as be aware of the experience of affect have on themselves.

Third, in counseling education, students develop self-awareness, learn new skills, build confidence, make better decisions, and gain insight into their choices. Observing others in a counseling process allowed students to unfreeze spontaneity, enact different roles, and evolve from cultural conservatism. Some people are taught to reveal only their public selves in a group and, consequently, in the beginning, students could be hesitant to reveal their true inner feelings [86]. They are more inclined to suppress their personal feelings and issues, especially in front of a group of unfamiliar persons. MEI is a self-administered scale, which allows individuals to test their experience of affect individually.

## Conclusions

This study provides important new insights into the psychometric properties of the MEI among university students. The MEI had a high degree of total internal consistency, acceptable reliability, and reasonable content validity. The proposed three-factor structure of the MEI was supported, as a PCA revealed three components: general, positive, and negative mirror effects. However, replication of the three-factor model in larger, more diverse samples is needed, together with an exploration of other validity methods, such as discriminant validity, to reinforce the internal validity of the MEI and support the measures of cross-cultural and external validity.

### Supplementary Information

**Additional file 1.** Study 1 questions for semi-structured interview.

**Additional file 2. **The mirror effect inventory.

## Data Availability

The datasets presented in this study can be found in online repositories. The names of the repository/repositories and accession number(s) can be found at: https://osf.io/teka7/files/osfstorage/63996053e4687409c79b4b1c.

## Abbreviations

CVI: Content validity index
CVR: Content validity ratio
KMO: Kaiser–Meyer–Olkin
MEI: Mirror Effects Inventory
PCA: Principal component analysis
