# Supplementary material for: Principal component analysis: development and initial validation of the mirror effects inventory
Source: BMC Psychol. 2023 Oct 31;11:363. doi: 10.1186/s40359-023-01397-8 (PMC10617217; doi:10.1186/s40359-023-01397-8)
Supplement: Supplementary file 1 — Additional file 1. Study 1 questions for semi-structured interview. [file 40359_2023_1397_MOESM1_ESM.docx]

**Additional file 1**

**Study 1 Questions for Semi-structured Interview**

**“Mirror Effects in Counseling Education Interview”**

***Questions for Respondents***

Demographic Information

1. In what year and semester did you undertake the course “Group Counseling/Group Counseling and Therapy”?
2. Are you a bachelor or master student? Which program are you in?
3. In what year will you or did you graduate from this program?
4. Do you have any work experience in individual counseling or group counseling? If yes, how long did you work in individual counseling or group counseling?

Experiences Gained from the Group Counseling Course

1. The Professor explained the theories and then used his own local real case audio recordings, video recordings, and real case live demonstration, which were real counseling cases of his former protagonists, to illustrate the skills used; in addition, role play and debriefing were implemented to analyze the function, process, and skills of different psychotherapies. In your opinion, which method(s) is/are the most appropriate approach from which to learn counseling? Why? Does it help? How?
2. When you counsel your clients, do you use anything learned from the course “Group Counseling/Group Counseling and Therapy”?
3. What is the most impressive thing in the course “Group Counseling/Group Counseling and Therapy”? Why?

Mirror Effects

*a)　Real case live demonstration*

1. Did you witness a real case live demonstration in this course? If yes, how did it make you feel?
2. Did you experience any differences before and after witnessing the real case live demonstration? How? Why?
3. Do you think a real case live demonstration is helpful for learning counseling skills? How? Why?

*b)　Audio case recordings or video case recordings*

1. Did you listen to audio recordings or watch video recordings in this course? If yes, how did it make you feel?
2. Did you experience any differences before and after listening to the audio recordings or watching the video recordings? How? Why?
3. Do you think listening and watching real case live demonstration recordings is helpful for improving your counseling skills? How? Why?

Professional Growth

1. In your opinion, when considering a real case live demonstration or audio case recordings or video case recordings, which teaching method is better for learning counseling? Why?
2. After taking “Group Counseling/Group Counseling and Therapy”, do you perceive any improvement in your counseling skills?
3. After taking “Group Counseling/Group Counseling and Therapy”, did you realize any differences in how you see things?
4. Have you used any lessons learned from the course “Group Counseling/Group Counseling and Therapy” in your workplace to solve problems? If yes, please give some examples. How do you use this learned lesson in your workplace?

Psychological Growth

1. Do you think this course is helpful for your personal growth? How? Why?
2. In your daily life, have you used any lesson learned to solve your own problems? How?
3. Have you used any lesson learned to help your relatives or friends to solve their problems? How?
