# Supplementary material for: Principal component analysis: development and initial validation of the mirror effects inventory
Source: BMC Psychol. 2023 Oct 31;11:363. doi: 10.1186/s40359-023-01397-8 (PMC10617217; doi:10.1186/s40359-023-01397-8)
Supplement: Supplementary file 2 — Additional file 2. The mirror effect inventory. [file 40359_2023_1397_MOESM2_ESM.docx]

**Additional file 2**

**The Mirror Effect Inventory**

Please read each statement and circle one answer, which indicates how much the statement applied to you. Please choose one answer for each question. There are no right or wrong answers.

|  | Strongly Disagree | Disagree | Neutral | Agree | Strongly Agree |
| --- | --- | --- | --- | --- | --- |
| 1. I feel in control of my problem. | 1 | 2 | 3 | 4 | 5 |
| 2. I execute the learned knowledge and/or skill to deal with significant events. | 1 | 2 | 3 | 4 | 5 |
| 3. I learn to handle significant events. | 1 | 2 | 3 | 4 | 5 |
| 4. I learn to solve interpersonal conflict. | 1 | 2 | 3 | 4 | 5 |
| 5. I discern the use of interpersonal skills. | 1 | 2 | 3 | 4 | 5 |
| 6. I develop a positive attitude to deal with difficulty. | 1 | 2 | 3 | 4 | 5 |
| 7. I use negative approach to handle problem. | 1 | 2 | 3 | 4 | 5 |
| 8. I recall past events. | 1 | 2 | 3 | 4 | 5 |
| 9. I associate similar experiences. | 1 | 2 | 3 | 4 | 5 |
| 10. I re-evaluate the pain of stressful events. | 1 | 2 | 3 | 4 | 5 |
| 11. I modify unrealistic expectations. | 1 | 2 | 3 | 4 | 5 |
| 12. I feel being ventilated. | 1 | 2 | 3 | 4 | 5 |
| 13. I express suppressed emotion. | 1 | 2 | 3 | 4 | 5 |
| 14. I gain support from my classmates. | 1 | 2 | 3 | 4 | 5 |
| 15. I gain support from my teachers. | 1 | 2 | 3 | 4 | 5 |
| 16. I regain vigor. | 1 | 2 | 3 | 4 | 5 |
| 17. I gain insight. | 1 | 2 | 3 | 4 | 5 |
| 18. I inspire new thinking. | 1 | 2 | 3 | 4 | 5 |
| 19. I find a clear goal. | 1 | 2 | 3 | 4 | 5 |
| 20. I gain new perspective. | 1 | 2 | 3 | 4 | 5 |
| 21. I develop self-reflection. | 1 | 2 | 3 | 4 | 5 |
| 22. I develop self-criticism. | 1 | 2 | 3 | 4 | 5 |
| 23. I evaluate myself. | 1 | 2 | 3 | 4 | 5 |
| 24. I gain self-knowledge. | 1 | 2 | 3 | 4 | 5 |
| 25. I indulge in problematic situation and/or imagery. | 1 | 2 | 3 | 4 | 5 |
| 26. I indulge in negative feelings. | 1 | 2 | 3 | 4 | 5 |
| 27. I have low self-confidence. | 1 | 2 | 3 | 4 | 5 |
| 28. I feel of low courage. | 1 | 2 | 3 | 4 | 5 |
| 29. I avoid solving the problem. | 1 | 2 | 3 | 4 | 5 |
| 30. I escape from the situation. | 1 | 2 | 3 | 4 | 5 |
| 31. I feel fear. | 1 | 2 | 3 | 4 | 5 |
| 32. I feel sorrow. | 1 | 2 | 3 | 4 | 5 |
| 33. I feel regret. | 1 | 2 | 3 | 4 | 5 |
| 34. I feel shame. | 1 | 2 | 3 | 4 | 5 |
| 35. I feel rejected. | 1 | 2 | 3 | 4 | 5 |
| 36. I blame other people. | 1 | 2 | 3 | 4 | 5 |
| 37. I blame myself. | 1 | 2 | 3 | 4 | 5 |
